# Chemical and Protein Characterization of Two Varieties of Chickpea (Cicer Arietinum): Costa 2004 and El Patrón

**DOI:** 10.3390/plants13152125

**Published:** 2024-08-01

**Authors:** Selene Pascual-Bustamante, Juan Carlos Raya-Pérez, César Leobardo Aguirre-Mancilla, Juan Gabriel Ramírez Pimentel, María Gabriela Vargas-Martínez, María Andrea Trejo-Márquez

**Affiliations:** 1 Posthaverst Laboratory of Plant Products, Faculty of Higher Studies Cuautitlan, Assimilation Technology Center Jiménez Cantú s/n, San Juan Atlamica, National Autonomous University of Mexico, Cuautitlan Izcalli 54729, Mexico; spluna27@cuautitlan.unam.mx (S.P.-B.); gvargasm@unam.mx (M.G.V.-M.); 2 Tecnológico Nacional de México/IT de Roque, Carretera Celaya-Juventino Rosas km 8, Celaya 38110, Mexico; juan.rp2@roque.tecnm.mx (J.C.R.-P.); cesar.am@roque.tecnm.mx (C.L.A.-M.); juan.rp1@roque.tecnm.mx (J.G.R.P.)

**Keywords:** legumes, amino acids, globulins, albumin, proteins

## Abstract

The objective of this study was to evaluate the chemical composition of two chickpea varieties, ‘Costa 2004’ and ‘El Patrón’, and to characterize their proteins to determine their technological potential for the food industry. For this purpose, chickpea samples of both varieties from the 2019 harvest region of Guanajuato, Mexico, were obtained and chemically characterized to determine the protein fractions using electrophoretic and amino acid profiling. The chickpea variety ‘Costa 2004’ contained 3% less protein and 7% less dietary fiber content than the variety ‘El Patrón’; whereas, the carbohydrate content of ‘Costa 2004’ was 4% greater. Additionally, the chickpeas demonstrated an antioxidant capacity ranging from 319 to 387 µMET/g and total phenol levels exceeding 500 mg/g. Among the protein fractions, globulins represented the highest proportion in both varieties of chickpea, at approximately 8.73 g/100 g (‘Costa 2004’) and 10.42 g/100 g (‘El Patrón’), followed by albumin, at approximately 1.24 g/100 g and 1.47 g/100 g, respectively. The chickpea proteins ranged in molecular weight between 100 and 25 kDa, with particularly strong signals in the albumin and globulin bands. Regarding the amino acid profile, histidine was predominant in both varieties. In conclusion, both varieties of chickpea have high nutritional value and broad potential for technological use in the food industry.

## 1. Introduction

Chickpea (*Cicer arietinum*) belongs to the Leguminosae family. It is an annual plant characterized by deep roots and hairy branched stems. Chickpea can be classified into two types based on its geographical distribution, Desi (originating in India) and Kabuli (originating in the Mediterranean) [1]. Some legumes, including chickpea (*Cicer arietinum*), have been studied only in the context of their traditional use as an animal feed; however, interest in their high protein content has increased, especially due to the shortage of protein for human consumption [2]. In addition to being a good source of protein, some varieties, such as Kabuli, contain low concentrations of antinutritional compounds like saponins, tannins, phytic acid, and trypsin inhibitors.

The main types of chickpeas grown in Mexico are those originating in the Mediterranean (France, Spain, and Italy) and Asian regions (India and Afghanistan). Chickpeas from the Mediterranean, known as Kabuli, are used for human consumption; they are characterized by their light color and large size. On the other hand, chickpeas from the Asian region are considered forage chickpea (Desi) and used mainly for animal feed; these chickpeas are smaller, wrinkled, and brown in color [1]. Although there are several studies on the use of legumes, little research is available on the use of chickpea. Chickpea and its flour are consumed largely in Asia as cooked chickpea, with curries, pasta, evening snacks, and energy supplements being examples. However, despite the high protein content, nutritional values, and health benefits, the value of protein in chickpea is rarely examined [3,4]. This legume has commercial relevance and has been consumed for its nutritional properties, especially its high protein content, representing a valuable food option [1].

The chemical composition of chickpea is characterized by a high content of fat and fiber and approximately 22% protein [5]. The protein content differs significantly depending on whether it is calculated with respect to the total mass of the dry chickpeas (17–22%) or with respect to the mass with the husk removed, in which case the protein content is higher (25.3–28.9%) [6]. Chickpea has good protein quality, meeting the requirements of essential amino acids for children from 2 to 5 years old, according to the OMS [7]. On the other hand, the digestibility of the protein contained in chickpeas can be from 76 to 78% [8].

Most of the proteins present in chickpea are storage proteins and are classified according to their solubility properties as albumins, globulins, and glutelins. Globulins represent approximately 70% of the total proteins in legumes (chickpeas, peas, and lentils). Albumins constitute between 10 and 20% of the total protein. Finally, glutelins range from 10 to 20% [9]. Since these proteins are mainly reserves, they have a low amount of sulfur-containing amino acids, such as methionine and cysteine. However, compared with those in cereals, the contents of lysine and arginine in chickpeas are high [10].

Chickpeas can be considered a functional food due to their high protein content and essential amino acid content. For instance, it has been shown that the consumption of proteins of plant origin can reduce mortality from cardiovascular diseases [11]. If the consumption of proteins of animal origin is reduced by 3%, mortality from conditions such as cancer or cardiovascular diseases would be reduced by up to 34%. This demonstrates the potential of the development of products using proteins of plant origin, such as chickpeas [12].

Chickpea proteins have higher bioavailability than those found in other legumes, positioning chickpeas as a promising substitute for the development of plant-based products. Therefore, it is crucial to carry out studies on different varieties of chickpea to obtain information on protein profile that will allow taking advantage of the nutritional properties of this legume and its potential applications in various technological options.

## 2. Results and Discussion

The chemical composition of the chickpeas studied is important information since the chemical properties of the chickpeas can potentially inform their technological potential for the development of new products.

The results from this study showed that chickpeas of the variety ‘El Patrón’ had approximately 3% higher protein content than the ‘Costa 2004’ variety (Table 1).

The protein content in Desi-type chickpeas tends to be greater than that in Kabuli-type beans [13]. This is related to the soil conditions and the type of cultivation, as well as the genetic and phenotypic characteristics of this variety. For this reason, the Desi variety is used as animal feed since a higher protein content is required for the growth of livestock. The chickpea ‘Costa 2004’ has approximately 5% greater carbohydrate content than the variety ‘El Patrón’, with a significant difference (*p* ≤ 0.05). Therefore, ‘Costa 2004’ is a good source of starch, which is the main carbohydrate present in legumes.

Chickpea is distinguished by its low glycemic index, which is attributable to its low content of amylose, a typical characteristic of legumes [13]. Another component of great interest is fiber, which is related to the development of functional foods. Therefore, the presence of fiber in a product increases its market value, and the chickpea variety ‘El Patrón’ possessed 5% more fiber than the variety ‘Costa 2004’, resulting in a statistically significant difference (*p* ≤ 0.05). According to the literature, dietary fiber content can reach up to 24.4%, primarily composed of non-cellulosic polysaccharides [14]. The high lipid content of both varieties, which exceeds the amount of lipids found in some other non-oleaginous legumes, is also important. The lipid content in chickpea can vary from 3.10 to 5.67% depending on the variety and growing conditions. The most prominent fatty acids in chickpea are linoleic and oleic acids [13].

In general, several factors influence the nutritional profiles of chickpea varieties, such as the chickpea variety, the environment, the nutritional status, and the agronomic practices and stress factors to which the plant is subjected [15].

Other nutrients associated with the chemical composition of chickpeas include minerals. Table 2 presents the results obtained from the characterization of these compounds.

In terms of mineral composition, chickpeas are recognized for being a significant source of phosphorus, followed closely by calcium. It is important to emphasize the low sodium content in chickpeas, establishing them as a viable dietary choice for individuals with hypertension. The importance of minerals in the immune system lies in their contribution to defense mechanisms, as they are essential components within cells and enzymes. Additionally, they function as inhibitors, activators, and regulators in human metabolism [16].

Other relevant compounds in chickpeas include those classified as non-nutritional; however, they may provide certain functional properties or health benefits. Table 3 presents the results obtained for these compounds.

The variety ‘El Patrón’ has a higher total phenolic content, approximately 100 mg/g more than compared to the chickpea ‘Costa 2004’. Some authors report levels reaching 470 mg/g, which includes both soluble phenols and those bound to other molecules. An important consideration from these authors is that heat treatments like cooking and roasting might reduce the overall phenolic compounds [17]. Among the phenols found in chickpeas, condensed tannins are notable, primarily located in the grain husk [14].

Phenolic compounds have diverse structural characteristics that confer free radical scavenging properties, which are closely related to antioxidant capacity [5]. There is a significant difference (*p* ≤ 0.05) in antioxidant capacity found between the two varieties. The variety ‘El Patrón’ has approximately 60 µmET/g greater antioxidant capacity content than the variety ‘Costa 2004’ . This finding corroborates previous research indicating that Desi-type chickpea varieties possess a greater capacity for scavenging free radicals compared to Kabuli types [16].

Chickpeas also contain saponins; notably the variety ‘Costa 2004’ had approximately twice the saponin content compared to the variety ‘El Patrón’. Saponins are considered non-nutritional compounds due to their ability to interfere with the absorption of nutrients such as minerals and fat-soluble vitamins [18]. However, it has been found that saponins can possess significant biological activity and potential utility in food development [19].

Similarly, trypsin inhibitors, which are compounds found in legumes, have long been considered non-nutritional because they reduce protein digestibility and absorption. However, recent studies suggest that they may have beneficial properties for humans and functional properties in food processing [20]. Analysis revealed that the variety ‘El Patrón’ demonstrated higher levels of trypsin inhibitors, and similar tendencies have been reported by other authors [16], who found greater trypsin inhibitor activity in Desi-type chickpea varieties (average 80.08 U/mg) compared to Kabuli types (average 43.83 U/mg).

The presence of aflatoxins in the chickpeas was also determined to establish whether they satisfied the safety requirements for use in the development of food products. No aflatoxins were detected in either chickpea variety; however, the chickpeas must be kept in appropriate storage conditions to prevent possible contamination.

### 2.1. Characterization of the Protein Fractions of Chickpeas

Most of the proteins present in chickpea are storage proteins, and they can be classified according to their constituent fractions. Globulins were the fraction that represented the highest percentage within both varieties of chickpea, 8.73 g/100 g in ‘Costa 2004’ and 10.42 g/100 g in ‘El Patrón’. Globulins are important storage proteins, and legumin is the predominant hexameric protein among them, containing amino acids such as methionine and cysteine [13]. The second largest fraction are the albumins, which represent approximately 1.24 and 1.47 g/100 g of the protein in ‘Costa 2004’ and in ‘El Patrón’, respectively. The albumins consist mainly of metabolic proteins, as well as enzymatic and non-enzymatic proteins [15]. Chickpea has 70% of trypsin inhibitors within albumins [21]. This distribution is due to the nature of the chickpea: the main proteins present in the chickpea are storage proteins, as reported by Roy et al. (2010), who also found them to consist mainly of globulins (70%) followed by albumin (10 to 20%). The prolamins content in the chickpea variety ‘El Patrón’ was 0.80 g/100 g, while in the variety ‘Costa 2004’ it was 0.84 g/100 g. There was no significant difference (*p* ≤ 0.05). The glutelins content in the chickpea variety ‘El Patrón’ was 1.13 g/100 g, whereas in the variety ‘Costa 2004’ the concentration of protein in the glutelin fractions was 0.66 g/100 g. Prolamines and glutelins make up less than 5% of the protein in legumes, which coincides with the findings in this work [15]. Notably, prolamines and glutelins are the least characterized protein fractions in chickpeas.

### 2.2. Electrophoretic Profiles of the Two Chickpea Varieties and Their Protein Fractions

**Figure 1:**
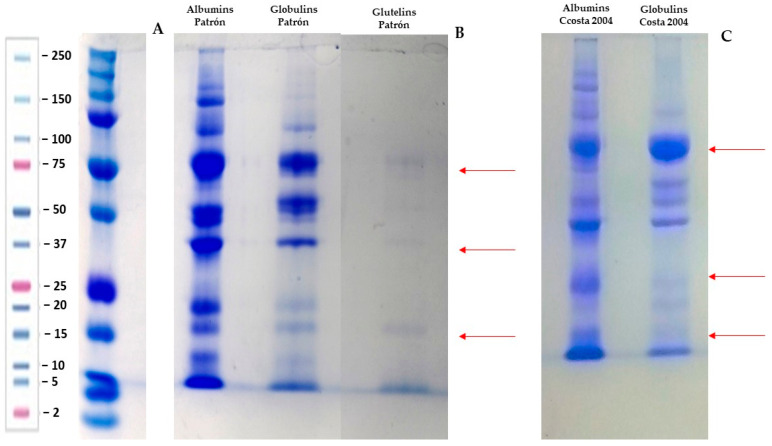
Polyacrylamide gel electrophoretic profile of the variety ‘Costa 2004’ and ‘El Patrón’. Chickpea protein; (**A**) Marker, (**B**) ‘El Patrón’ and (**C**) ‘Costa 2004’.

Once the protein fractions of the two varieties were obtained, their electrophoretic profiles were evaluated, both for the total proteins contained in the chickpeas and for each protein fraction. The electrophoretic profiles of the two varieties of chickpeas ‘Costa 2004’ and ‘El Patrón’ were evaluated for each protein fraction (Figure 1).

In the electrophoretic profile of the ‘El Patrón’ chickpea proteins (Figure 1B), two main bands were observed at 100 and 75 kDa. In the albumin fraction, the distribution of proteins ranged from 100 to 15 kDa, indicating the presence of legumin and vicilin [22]. Proteins with weights less than 25 kDa are considered to be low-molecular-weight proteins especially albumin (2S) [6]. In the globulin fraction, bands were observed at 75, 50, 37 25, and 20 kDa. On the other hand, no bands were observed in the prolamine fraction. In the glutelin fraction, bands were visible at 75, 50, and 15 kDa but were not sharp. In beans (*Phaseolus vulgaris*) from Mexico varieties (Bayo Berrendo, and Patzcuareño), it has been reported that the bands observed for the albumin and globulin fractions are similar, which is consistent with the results obtained in this work [23].

The fraction of albumin contained in the protein in the variety ‘Costa 2004’ had weights between 75 and 25 kDa (Figure 1C). A previous study detected chickpea protein extract bands ranging from 92 to 12 kDa, characteristic of legumes [6], indicating the presence of legumin (11S), vicilin, and albumin (2S) in the albumin fraction [6]. In the globulin fraction, bands were observed at 75, 50, and 37 kDa and corresponded to vicilin (7S) and legumin (11S), respectively. No bands were observed in the prolamine or glutelin fractions, and it was reported low intensity in the prolamine and glutelin bands as well [6]. In parota seed protein (*Enterolobium cyclocarpum*), it is has been reported a wide distribution in the albumin protein fraction between 180 and 10 kDa. In this study, the largest number of bands appeared in the albumin fraction, with a wide distribution of molecular weights [24].

### 2.3. Amino Acid Profiles of the Proteins in the Two Chickpea Varieties

Chickpeas are a rich source of protein, presenting an advantage due to the richness of highly digestible amino acids, which can represent 36% of their composition [25].

**Figure 2:**
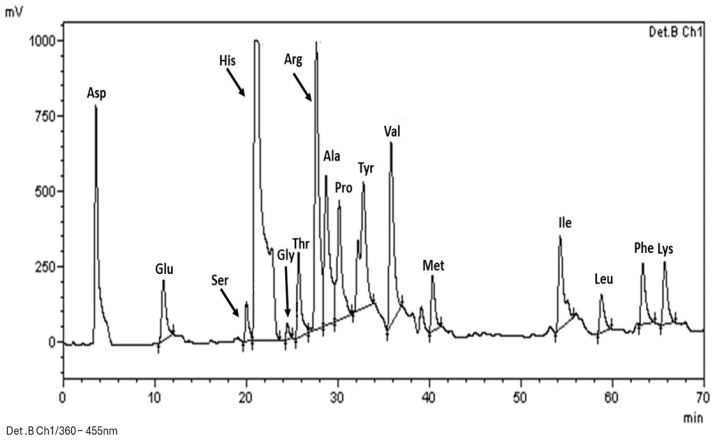
Identification of amino acids present in the chickpea variety ‘Costa 2004’. The amino acids identified are alanine (Ala), arginine (Arg), aspartic acid (Asp), cysteine, glutamic acid (Glu), glycine (Gly), histidine (His), isoleucine (Ile), leucine (Leu), lysine (Lys), methionine (Met), phenylalanine (Phe), proline (Pro), serine (Ser), threonine (Thr), tyrosine (Tyr), and valine (Val).

Each amino acid signal in the chromatogram of the variety ‘Costa 2004’ (Figure 2) was identified using two methods: (1) comparing the retention times to those in the chromatogram of the mixture of amino acid standards and (2) adding selected standards to the test samples and observing the increase in peak height.

The results showed that chickpeas contain various essential amino acids, including arginine (Arg), phenylalanine (Phe), leucine (Leu), isoleucine (Ile), lysine (Lys), methionine (Met), threonine (Thr), and valine (Val), but not tryptophan, which was degraded during the hydrolysis of the sample, or cysteine signal, which was expected since various authors have reported that chickpea is low in this amino acid. Previous work has reported that methionine was present at low concentrations (0.8%) in the chickpea protein fraction, which is consistent with the results of this work [6]. Notably, the signals corresponding to aspartic acid, histidine, and arginine are more intense than those corresponding to the other amino acids present.

These data support the previous reports that aspartic acid constituted up to 10% of the total protein in chickpea, while arginine constituted approximately 7% [16].

**Figure 3:**
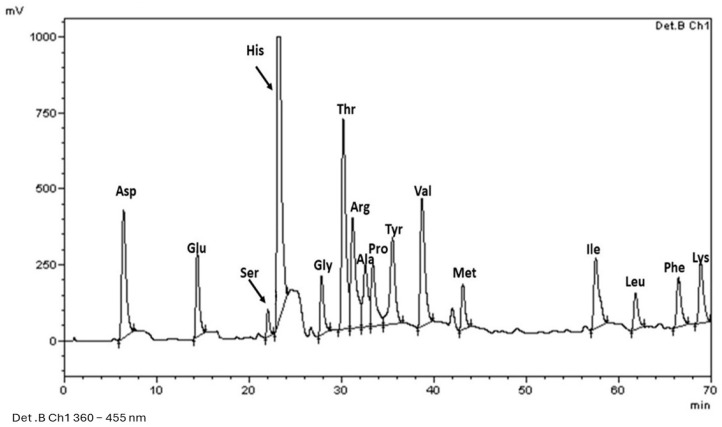
Identification of amino acids present in the chickpea variety ‘El Patrón’. The amino acids identified are alanine (Ala), arginine (Arg), aspartic acid (Asp), cysteine, glutamic acid (Glu), glycine (Gly), histidine (His), isoleucine (Ile), leucine (Leu), lysine (Lys), methionine (Met), phenylalanine (Phe), proline (Pro), serine (Ser), threonine (Thr), tyrosine (Tyr), and valine (Val).

In the chickpea variety ’El Patrón’ (Figure 3), notably, histidine produced a more intense signal than the other amino acids in chickpeas. On the other hand, it has been reported that the combined content of aspartic acid, glutamic acid, and arginine can reach 42.16 g/100 g of protein in chickpeas [6,26].

In the previously studied amino acid profile of chickpea, it was found that at least 30% of the protein in chickpea consists of essential amino acids [6]. This finding is highly relevant: although the amino acid content of chickpeas was not quantified in this study, the protein profile of chickpeas is known to include essential amino acids, which indicates substantial nutritional value. Notably, although some of the amino acids present in chickpea are not essential, they are linked to the functional properties of proteins due to the hydrophilic and hydrophobic groups present in their structure. These components can influence the surface hydrophobicity, electrostatic interactions, and stability of the proteins [6]. Both varieties presented greater intensity in the histidine signal. Recent studies have shown that histidine is a nutritional requirement for adults, requiring a consumption of 0.7 g/day in the diet [27].

In this experiment, tryptophan was not detected since it is a sulfur-containing amino acid, which is found in limited quantities in chickpeas, primarily because it is present in the polyamine fraction [28]. As observed, chickpeas show low levels of this protein fraction, with concentrations measuring less than 1 g/100 g of protein in both varieties.

Chickpeas are abundant in albumins and globulins, which are rich in essential amino acids such as lysine and threonine [29]. These protein fractions are crucial for creating foams and emulsions, contributing to the significant technological potential of chickpeas for protein utilization. This research demonstrates that chickpeas primarily contain aromatic amino acids, including lysine, leucine, and isoleucine, meeting the OMS prescribed amino acid requirements for preschool children [29].

### 2.4. Characterization of Techno-Functional Properties of Chickpea Flour

Chickpeas contain a variety of compounds in their composition that contribute to their diverse technological properties, making them valuable for developing new products. The results from the analyses of chickpea flours are summarized in Table 4.

The water absorption capacity is a techno-functional property directly related to protein content and protein–water interaction [30]. The chickpeas of both varieties showed over 1 g of water absorbed per gram of flour with a significant difference (*p* ≤ 0.05). Water absorption values in chickpea flour have been reported at 1.83 g H_2_O/g, indicating that this property increases during soaking or cooking due to protein denaturation and starch gelatinization.

Oil absorption capacity was shown to be high in ‘Costa 2004’ chickpeas, although it was lower than the reported literature value of 1.16 g of oil/g [5]. This property is associated with fiber content and hydrophobic interactions of proteins [31].

The chickpea flours exhibit a foaming capacity of over 60% due to two primary factors. The high albumin content and the presence of saponins contribute to form stable foams. However, exposing chickpeas to certain processes such as soaking or cooking reduces foaming capacity, mainly due to protein denaturation [31].

Emulsifier capacity did not exceed 50%. This property is associated with the interaction between proteins and starch, resulting in an enlargement of particle sizes due to the size of starch granules. The chickpea has a better emulsifier capacity compared to those of soy and pea concentrates. This could be attributed to the specific protein types and their respective flexibility [26,29].

The gelation capacity was also tested by diluting flours in water. The results indicated that gel formation commenced with a 12% flour dilution in both chickpea varieties. This property is associated with the plasticity, viscosity, and elasticity in foods, and previous studies have demonstrated the formation of firm gels from solutions ranging between 13 and 20% [5].

## 3. Materials and Methods

### 3.1. Biological Materials

For the development of the project, two types of chickpeas were used: the Kabuli type ‘Costa 2004’ variety and the Desi variety ‘El Patrón’ both from the 2019 harvest in Celaya, Guanajuato, Mexico. During the experiments, the chickpeas were kept in hermetically sealed flasks at room temperature.

### 3.2. Chemical Characterization of Chickpea 

The chickpea variety ‘Costa 2004’ was chemically characterized to evaluate parameters such as moisture content, lipid content, ash content, fiber content, and protein content [32] and carbohydrates were calculated by the difference with the other components. Within the chemical composition, the presence of minerals such as Na^+^, Ca^+^, Mg^+^, and K^+^ was evaluated [32]. Other parameters to evaluate included total phenolic content [33], antioxidant capacity [34], the presence of saponins [35], and trypsin inhibator levels [20]. The presence of aflatoxins was evaluated using monoclonal antibody columns and fluorometric detection (Vicam) [36] to determine the safety of the chickpeas.

### 3.3. Protein Profile by Solubility 

Protein fractionation was performed by the Osborne method [37], which consists of separating the proteins based on their solubility in the following solvents: purified water (albumins), 0.5 M NaCl (globulins), 70% ethanol (prolamines), and 0.1 N NaOH (glutelins). Each fraction was quantified using the Bradford method [38]. The results are expressed as a g/100 g of the protein contained in the chickpeas.

### 3.4. Electrophoretic Profile 

Proteins were separated on a 10% polyacrylamide gel with a tricine buffer solution. A vertical electrophoresis chamber was used. The gels were stained with Coomassie blue and fixed with a methanol–acetic acid mixture for 12 h [39].

### 3.5. Identification of Amino Acids in Chickpeas

Hydrolysis. For the identification of amino acids, the proteins present in the chickpeas were hydrolyzed. The sample was treated with 6 N HCl at 120 °C for 24 h. Subsequently, the pH was adjusted to 2.2 by the addition of 8 N NaOH. The samples were stored in amber flasks for later use [32].

Derivatization. For derivatization, 5 µL samples of the hydrolyzed proteins were placed in vials, followed by the addition of 30 µL of distilled water and 25 µL of OPA/2ME (125 mg of orthophthaldehyde, 2 mercaptanol, and tetrahydrofuran diluted in 1 mL of methanol with 50 µL of mercaptanol). The mixture was brought up to a volume of 5 mL with a saturated solution of sodium borate (pH 10). After shaking for 1 min, the samples were injected into the equipment within the next 2 min.

Separation. Separation was performed with high-performance liquid chromatography (HPLC) equipment (Brand: Shimadzu/Japan, Model: CTO-10A). The derivatized samples were injected (5 µL) into an AccQ-Tag amino acid C_18_ column (Waters, 60 Å, 4 µm, 3.9 mm × 150 mm) at a flow rate of 1 mL/min using an eluent gradient. Eluent A consisted of 0.08 M phosphate buffer (pH 7.2), while eluent B was a 55:45 methanol-phosphate buffer (0.08 M, pH 7.2).

Identification. A mixture of standards composed of alanine (Ala), arginine (Arg), aspartic acid (Asp), cysteine, glutamic acid (Glu), glycine (Gly), histidine (His), isoleucine (Ile), leucine (Leu), lysine (Lys), methionine (Met), phenylalanine (Phe), proline (Pro), serine (Ser), threonine (Thr), tyrosine (Tyr), and valine (Val) from Fluka (09418) were used at a concentration of 0.01 M in 0.1 M hydrochloric acid solution. A fluorescence detector (Brand: Shimadzu, Model: RF-10Ax) was used for identification.

### 3.6. Characterization of Techno-Functional Properties of Chickpea Flour

Chickpea beans were ground to obtain two flours with particle sizes of 0.841 mm and 0.420 mm in both varieties. Each flour was evaluated for its techno-functional properties.

Water absorption capacity: In 2.5 g of chickpea flour, 5 mL of distilled water was added and mixed for 1 min. The sample was then incubated at room temperature for 30 min and subsequently centrifuged at 1000 rpm for 30 min. The supernatant was decanted, and the mass of the wet pellet was measured to determine the water per mass of the dry pellet [40].

Oil absorption capacity: The 0.5 g sample was mixed with 5 mL of oil. It was rested for 30 min at room temperature and subsequently centrifuged at 3000 rpm for 30 min. Finally, the supernatant was discarded. The results were reported in g oil/g of sample [31].

Foaming capacity: The 2 g sample was mixed in 100 mL of distilled water and then homogenized for 2 min. The foam capacity was calculated as a percentage [31].

Gelation capacity: The gelation was measured in triplicate in suspensions of the sample at concentrations of 4, 8, 12, 16, and 20% in distilled water. Subsequently, the samples were incubated in a water bath at 100 °C for 1 h, followed by immersion in an ice bath for 1 h [5].

Emulsifier capacity: In the conducted procedure, 0.5 g of the sample was combined with 2.5 mL of distilled water and 2.5 mL of oil. Subsequently, the emulsion was centrifuged at 3000 rpm for 1 h, after which the emulsified layer was gauged in relation to the total volume [31].

These evaluations aimed to assess the technological potential for utilizing the two distinct chickpea varieties.

### 3.7. Statistical Analysis

The statistical analysis of the chemical composition involved a comparison of means with a confidence interval of 95%. For protein fractionation, multifactorial analysis was performed with Tukey’s multiple range test, and the significance level for both analyses was 0.05. Analyses were performed using SPSS version 20.

## 4. Conclusions

The chickpeas studied represent an important source of protein, especially the variety ‘El Patrón’, which had a protein content approximately 3% higher than that of the variety ‘Costa 2004’. In terms of protein composition, the globulin fraction was the most abundant in both chickpea varieties, followed by the albumin fraction. The molecular weights of the proteins indicate the presence of vicilin and legumin, with weights ranging from 75 to 25 kDa. The chickpea varieties studied both contained all essential amino acids except tryptophan in their profiles. Histidine was especially abundant in both varieties. In the variety ‘Costa 2004’, aspartic acid and arginine were particularly prominent, while in the variety ‘El Patrón’, threonine was the second most abundant after histidine. Chickpea has functional properties applicable to the development of functional foods, such as plant-based beverages, egg substitutes, or analogues of dairy products. In conclusion, both chickpea varieties are economical resources with potential for the technological development of products rich in proteins of plant origin, which can replace those of animal origin, generating an alternative for consumption. In addition, this crop is a viable option for areas with dry weather as chickpeas are low-water-consuming and drought resistant.

## Figures and Tables

**Table 1:** Chemical compositions of the chickpea varieties ‘El Patrón’ and ‘Costa 2004’, g per 100 g of product.

Component	‘El Patrón’	‘Costa 2004’
Carbohydrates (g)	52.42 ± 1.71 a	59.64 ± 0.94 b
Protein (g)	14.08 ± 0.85 b	11.45 ± 0.56 a
Moisture (g)	10.11 ± 0.30 b	8.41 ± 0.14 a
Fat (g)	2.84 ± 0.18 a	5.99 ± 0.04 b
Dietary Fiber (g)	17.21 ± 0.99 b	10.95 ± 0.57 a
Ash (g)	3.33 ± 0.03 a	3.54 ± 0.01 b

Different letters (a, b) in each column indicate a significant difference (*p* ≤ 0.05) between grain varieties.

**Table 2:** Evaluation of minerals in the two chickpea varieties.

Mineral	‘El Patrón’	‘Costa 2004’
Ca^+^	0.201 ± 0.00 a	0.162 ± 0.00 b
Mg^+^	0.052 ± 0.00 b	0.031 ± 0.00 a
P^+^	1.137 ± 0.00 a	1.263 ± 0.00 b
Na^+^	0.013 ± 0.00 a	0.014 ± 0.00 b

Different letters (a, b) in each column indicate a significant difference (*p* ≤ 0.05) between grain varieties.

**Table 3:** Evaluation of bioactive and non-nutritional compounds in chickpea beans.

Component	‘El Patrón’	‘Costa 2004’
Total phenols (mg/g)	591.82 ± 18.52 b	494.53 ± 23.74 a
Antioxidant capacity (µmET/g)	387.54 ± 26.68 b	319.91 ± 8.90 a
Saponins (mg/g)	134.98 ± 4.91 a	307.06 ± 4.42 b
Trypsin inhibitor (U/mg)	21.76 ± 0.47 b	18.41 ± 1.42 a

Different letters (a, b) in each column indicate a significant difference (*p* ≤ 0.05) between grain varieties.

**Table 4:** Techno-functional properties of chickpea beans.

Property	‘El Patrón’	‘Costa 2004’
Water absorption capacity (g of H_2_O/g)	1.44 ± 0.01 b	1.28 ± 0.04 a
Oil absorption capacity (g of oil/g)	0.77 ± 0.03 a	1.04 ± 0.02 b
Foaming capacity (%)	64.53 ± 1.26 a	63.96 ± 1.34 a
Emulsifier capacity (%)	46.20 ± 0.44	47.45 ± 0.84 a

Different letters (a, b) in each column indicate a significant difference (*p* ≤ 0.05) between grain varieties.

## Data Availability

The original contributions presented in the study are included in the article, further inquiries can be directed to the corresponding authors.

## References

[1] Aguilar Raymundo V.G., Vélez-Ruiz J.F. (2013). Propiedades nutricionales y funcionales del garbanzo (*Cicer arietinum* L.). TSIA.

[2] Serventi L., Ferranti P. (2023). Functional Ingredients of Chickpea. Sustainable Food Science—A Comprehensive Approach.

[3] Altaf U., Zameer Hussain S., Qadri T., Aafiya Ishrat S., Kanojia V. (2020). Optimization of Extrusion Process for Development of Nutritious Snacks using Rice and Chickpea Flour. J. Sci. Ind. Res..

[4] Acevedo Martinez K.A., Yang M.M., Gonzalez de Mejia E. (2021). Technological properties of chickpea (*Cicer arietinum*): Production of snacks and health benefits related to type-2 diabetes. Compr. Rev. Food Sci. Food Saf..

[5] Ponce-Fernández N.E., Pollorena-López G., Rosas-Domínguez C., López-Peñuelas M.V., Osuna-Izaguirre S.C. (2017). Composición química, características funcionales y capacidad antioxidante de formulaciones de garbanzo (*Cicer arietinum* L.) blanco Sinaloa 92. Agrociencia.

[6] Soto-Madrid D., Pérez N., Gutiérrez-Cutiño M., Matiacevich S., Zúñiga R.N. (2023). Structural and Physicochemical Characterization of Extracted Proteins Fractions from Chickpea (*Cicer arietinum* L.) as a Potential Food Ingredient to Replace Ovalbumin in Foams and Emulsions. Polymers.

[7] FAO, WHO (1973). Energy and Protein Requirements.

[8] Rahman M.S., Sana N.K., Hasan M.M., Huque M.E., Shaha R.K. (2008). Enzyme activities and degradation of nutrients in chickpea (*Cicer arietinum* L.) seeds during germination. J. Biosci..

[9] Roy F., Boye J.I., Simpson B.K. (2010). Bioactive proteins and peptides in pulse crops: Pea, chickpea and lentil. Food Res. Int..

[10] Duranti M. (2006). Grain legume proteins and nutraceutical properties. Fitoterapia.

[11] Chen Z., Glisic M., Song M., Aliahmad H.A., Zhang X., Moumdjian A.C., Gonzalez-Jaramillo V., Van der Schaft N., Bramer W.M., Ikram M.A. (2020). Dietary protein intake and all-cause and cause-specific mortality: Results from the Rotterdam Study and a meta-analysis of prospective cohort studies. Eur. J. Epidemiol..

[12] Craig W.J., Mangels A.R., American Dietetic Association (2010). Postura de la Asociación Americana de Dietética: Dietas vegetarianas. J. Am. Diet. Assoc..

[13] Grasso N., Lynch N.L., Arendt E.K., O’Mahony J.A. (2022). Chickpea protein ingredients: A review of composition, functionality, and applications. Compr. Rev. Food Sci. Food Saf..

[14] Niño-Medina G., Muy-Rangel D., Garza-Juárez A.D.J., Vázquez-Rodríguez J.A., Méndez-Zamora G., Urías-Orona V. (2017). Composición nutricional, compuestos fenólicos y capacidad antioxidante de cascarilla de garbanzo (*Cicer arietinum*). ALAN.

[15] Shevkani K., Singh N., Chen Y., Kaur A., Yu L. (2019). Pulse proteins: Secondary structure, functionality and applications. J. Food Sci. Technol..

[16] Kaur K., Grewal S.K., Gill P.S., Singh S. (2019). Comparison of cultivated and wild chickpea genotypes for nutritional quality and antioxidant potential. J. Food Sci. Technol..

[17] Godrich J., Rose P., Muleya M., Gould J. (2023). The effect of popping, soaking, boiling and roasting processes on antinutritional factors in chickpeas and red kidney beans. Int. J. Food Sci. Technol..

[18] Antoine T., Georgé S., Leca A., Desmarchelier C., Halimi C., Gervais S., Aupy F., Marconot G., Reboul E. (2022). Reduction of pulse ‘antinutritional’ content by optimizing pulse canning process is insufficient to improve fat-soluble vitamin bioavailability. Food Chem..

[19] Wang Y., Ma Y., Tao L., Zhang X., Hao F., Zhao S., Han L., Bai C. (2022). Recent Advances in Separation and Analysis of Saponins in Natural Product. Separations.

[20] Nagl N., Sinkovic L., Savic A., Isakov M., Hasanaklou H.T., Pipan B., Jeromelal A.M. (2023). Trypsin inhibitor activity in grass pea seeds (*Lathyrus sativus* L.). Ratar. Povrt..

[21] Clemente A., Vioque J., Sánchez-Vioque R., Pedroche J., Bautista J., Millán F. (2000). Factors affecting the in vitro protein digestibility of chickpea albumins. J. Sci. Food Agric..

[22] Chang Y.W., Alli I., Molina A.T., Konishi Y., Boye J.I. (2012). Isolation and characterization of chickpea (*Cicer arietinum* L.) Seed Protein Fractions. Food Bioproc. Technol..

[23] Raya-Pérez J.C., Gutiérrez-Benicio G.M., Ramírez Pimentel J.G., Covarrubias-Prieto J., Aguirre-Mancilla C.L. (2014). Caracterización de proteínas y contenido mineral de dos variedades de frijol native de México. Agro. Mesoam..

[24] Espitia Orozco F.J., Negrete Toledo A.G., Ordoñez Acevedo L.G., León Galván M.F. (2016). Caracterización de las proteínas de reserva de la semilla de parota (*Enterolobium cyclocarpum*)-Resumen. Investig. Des Cien. Tec. Alim..

[25] Herrera Flores S., Moreno Contreras M.G., Licea De Anda E.M., Castro Alda A.A., Medina Haro A. (2021). Análisis bromatológicos y funcionales de tres variedades comerciales de garbanzo (*Cicer arietinum* L.). Braz. J. Anim. Environ. Res..

[26] Ghribi A.M., Gafsi I.M., Blecker C., Danthine S., Attia H., Besbes S. (2015). Effect of drying methods on physico-chemical and functional properties of chickpea protein concentrates. J. Food Eng..

[27] Brosnan M.E., Brosnan J.T. (2020). Histidine Metabolism and Function. J. Nutr..

[28] Bai T., Nosworthy M.G., House J.D., Nickerson M.T. (2018). Effect of tempering moisture and infrared heating temperature on the nutritional properties of desi chickpea and hull-less barley flours, and their blends. Food Res. Int..

[29] Boukid F. (2021). Chickpea (*Cicer arietinum* L.) protein as a prospective plant-based ingredient: A review. Int. J. Food Sci. Technol..

[30] Serna-Cock L., Pabón-Rodríguez O.V., Quintana-Moreno J.D. (2019). Effects of the ionic force and time of soaking of dry legumes on their tecnofuncional properties. Inf. Tecnológica.

[31] García Pacheco Y., Cabrera Mercado D., Ballestas Santos J.A., Campo Arrieta M.J. (2019). Efecto de diferentes tratamientos térmicos sobre las propiedades tecfuncionales de la harina de fríjol blanco (*Phaseolus lunatus* L.) y la determinación de su potencial uso agroalimentario. Inge Cuc.

[32] AOAC International (2019). Official Methods of Analysis of AOAC International.

[33] Rochín-Medina J.J., Navarro-Cortez R.O., Tovar-Jiménez X., Quiñones-Reyes G., Ayala-Luján J.L., Aguayo-Rojas J. (2021). Contenido de compuestos fenólicos y capacidad antioxidante de variedades de frijol sembradas en el estado de Zacatecas. Acta Univ..

[34] Fogliano V., Verde V., Randazzo G., Ritieni A. (1999). Method for measuring antioxidant activity and its application to monitoring the antioxidant capacity of wines. J. Agric. Food Chem..

[35] Guzmán B., Cruz D.L., Alvarado J.A., Mollinedo P. (2013). Cuantificación de saponinas en muestras de Cañihua *Chenopodium pallidicaule* Aellen. Rev. Bol. Quim..

[36] Vicam (1999). AflaTest WB SR Instruction Manual.

[37] Moscoso-Mujica G., Zavaleta A., Mujica Á., Santos M., Calixto R. (2017). Fraccionamiento y caracterización electroforética de las proteínas de la semilla de kañihua (*Chenopodium pallidicaule* aellen). Rev. Chil. Nutr..

[38] Bradford M.M. (1976). A Rapid and Sensitive Method for the Quantitation of Microgram Quantities of Protein Utilizing the Principle of Protein-Dye Binding. Anal. Biochem..

[39] Rahman M., Liu L., Barkla B.J. (2021). A single seed protein extraction protocol for characterizing brassica seed storagproteins. Agronomy.

[40] Vargas-Salazar T.A., Wilkinson K.A., Urquiaga-Zavaleta J.M., Rodríguez-Zevallos A.R. (2020). Physicochemical characterization of ÑuÑa bean (*Phaseolus vulgaris* L.) protein extract. Vitae.

